# Adrenal βarrestin1 targeting for tobacco–associated cardiac dysfunction treatment: Aldosterone production as the mechanistic link

**DOI:** 10.1002/prp2.497

**Published:** 2019-06-18

**Authors:** Maria E Solesio, Erna Mitaishvili, Anastasios Lymperopoulos

**Affiliations:** ^1^ Department of Basic Sciences New York University New York New York; ^2^ Laboratory for the Study of Neurohormonal Control of the Circulation, Department of Pharmaceutical Sciences Nova Southeastern University College of Pharmacy Fort Lauderdale, Florida

**Keywords:** adrenal cortex, aldosterone, angiotensin II, nicotine, tobacco–related heart disease, βarrestin

## Abstract

Tobacco kills 6 million people annually and its global health costs are continuously rising. The main addictive component of every tobacco product is nicotine. Among the mechanisms by which nicotine, and its major metabolite, cotinine, contribute to heart disease is the renin‐angiotensin‐aldosterone system (RAAS) activation. This increases aldosterone production from the adrenals and circulating aldosterone levels. Aldosterone is a mineralocorticoid hormone with various direct harmful effects on the myocardium, including increased reactive oxygen species (ROS) generation, which contributes significantly to cardiac mitochondrial dysfunction and cardiac aging. Aldosterone is produced in the adrenocortical zona glomerulosa (AZG) cells in response to angiotensin II (AngII), activating its type 1 receptor (AT_1_R). The AT_1_R is a G protein‐coupled receptor (GPCR) that leads to aldosterone biosynthesis and secretion, via signaling from both G_q/11_ proteins and the GPCR adapter protein βarrestin1, in AZG cells. Adrenal βarrestin1 is essential for AngII–dependent adrenal aldosterone production, which aggravates heart disease. Since adrenal βarrestin1 is essential for raising circulating aldosterone in the body and tobacco compounds are also known to elevate aldosterone levels in smokers, accelerating heart disease progression, our central hypothesis is that nicotine and cotinine increase aldosterone levels to induce cardiac injury by stimulating adrenal βarrestin1. In the present review, we provide an overview of the current literature of the physiology and pharmacology of adrenal aldosterone production regulation, of the effects of tobacco on this process and, finally, of the effects of tobacco and aldosterone on cardiac structure and function, with a particular focus on cardiac mitochondrial function. We conclude our literature account with a brief experimental outline, as well as with some therapeutic perspectives of our pharmacological hypothesis, that is that adrenal βarrestin1 is a novel molecular target for preventing tobacco–induced hyperaldosteronism, thereby also ameliorating tobacco–related heart disease development.

**Abbreviations**
AngII: angiotensin II
AT_1_R: angiotensin II type I receptor
AZG: adrenocortical zona glomerulosa
CHF: chronic heart failure
DAG: diacylglycerol
ENDS: electronic nicotine delivery system
ETC: Electron Transport Chain
GPCR: G protein‐coupled receptor
IP_3_: 1`, 4`, 5`‐inositol trisphosphate
MAPK: mitogen‐activated protein kinase
mPTP: Mitochondrial Permeability Transition Pore
MR: mineralocorticoid receptor
mtDNA: mitochondrial DNA
PLC: phospholipase C
polyP: polyphosphate
PTH: parathyroid hormone
RAAS: renin‐angiotensin‐aldosterone system
ROS: reactive oxygen species
StAR: Steroidogenic Acute Regulatory

## 1 INTRODUCTION: TOBACCO AND ALDOSTERONE

Aldosterone is one of a number of hormones with detrimental functions for the failing heart, whose circulating levels are elevated in chronic heart failure (CHF), contributing significantly to its morbidity and mortality.^1^, ^2^, ^3^, ^4^ Aldosterone`s detrimental actions on the heart include (but are not limited to) cardiac hypertrophy, fibrosis, and increased inflammation and oxidative stress, all of which result in adverse cardiac remodeling and progressive loss of cardiac function and performance.^1^, ^2^, ^3^, ^4^ Accordingly, plasma aldosterone levels are a marker of CHF severity^5^ and aldosterone antagonists, such as spironolactone and its newer congener eplerenone, have well–documented beneficial effects in CHF and constitute a significant segment of the CHF pharmacotherapeutic regimen.^6^, ^7^

Aldosterone is also the final hormone produced upon activation of the renin‐angiotensin‐aldosterone system (RAAS) axis.^8^ Together with angiotensin II (AngII), which is one of the most potent physiological stimuli for its production and secretion from the adrenal glands, aldosterone exerts a variety of effects throughout the cardiovascular system, normally aiming at maintaining renal perfusion and correcting electrolyte (Na^+^, K^+^) and blood volume imbalances.^8^ In the presence of heart disease however, especially under CHF, aldosterone (and AngII) is overproduced and markedly elevated in the circulation, and its cardiovascular actions become maladaptive, hampering cardiac function, indirectly, via blood pressure (cardiac afterload) elevation, but also via direct actions in the heart, resulting in adverse remodeling (eg hypertrophy, fibrosis, oxidative stress, inflammation, etc).^9^, ^10^, ^11^

The main tobacco compound nicotine, and cotinine, its major metabolite in humans^12^, have been reported to activate the RAAS axis upon chronic use in humans (ie in chronic smokers)^13^, ^14^, ^15^, ^16^, ^17^; reviewed in ref 18. Of course, nicotine is the main addictive component in tobacco products but is not the only harmful ingredient in tobacco by any means. Tar and other polycyclic aromatic hydrocarbon compounds, polyethylene glycol (used commonly in electronic nicotine delivery systems), and myriad other substances contained in every single tobacco product on the market can also cause significant cardiovascular harm.^19^ However, the effects of tobacco on RAAS have so far been studied only in relation to nicotine. Given the well–established harmful effects of both AngII and aldosterone in the heart and blood vessels, nicotine–induced RAAS activation is bound to contribute to the development of heart disease, specifically of CHF, by nicotine and cotinine in chronic tobacco smokers. However, the specific actions of these tobacco compounds in the modulation of the production of adrenocortical aldosterone under physiological conditions have not been studied.

Another emerging area of tobacco research, currently under intense investigation, is that of the biological effects of e‐cigarettes and other electronic nicotine delivery systems (ENDS) used for vaping. These devices are battery powered units that vaporize a liquid, most commonly containing glycerol, propylene glycol, flavoring and, of course, nicotine.^20^ Heavily promoted by the industry, with fierce marketing campaigns targeted mainly at the most socially vulnerable and easily influenced populations, such as adolescents and young adults, they are growing fast in popularity also because they are promoted as safer nicotine products compared to traditional tobacco.^21^, ^22^ The latter contain tar and other harmful chemicals, which ENDS lack. Also, e‐cigarettes purportedly do not cause secondhand smoke and can deliver less nicotine than traditional cigarettes, so they can be used as nicotine replacement therapy.^20^, ^23^ Nevertheless, there is already a substantial amount of evidence that these products can be equally (if not even more) harmful to the cardiovascular system than traditional tobacco. Like classic tobacco smoking, ENDS can also increase sympathetic nervous system activity, oxidative stress and inflammation, endothelial dysfunction, and platelet activation, leading to cardiac arrhythmias, atherosclerosis and plaque instability, thrombosis and acute ischemia, etc (as reviewed recently by 23). The effects of e‐cigarettes on RAAS activity and specifically on aldosterone levels are presently not known. Given the similarity between their cardiovascular effects and those of traditional tobacco smoking, it is quite plausible that ENDS–derived nicotine also activates the RAAS axis and elevates circulating aldosterone levels, exactly as the nicotine delivered by traditional tobacco products does.

## 2 ROLE OF βARRESTIN1 IN ADRENAL ALDOSTERONE PRODUCTION

**Figure 1:**
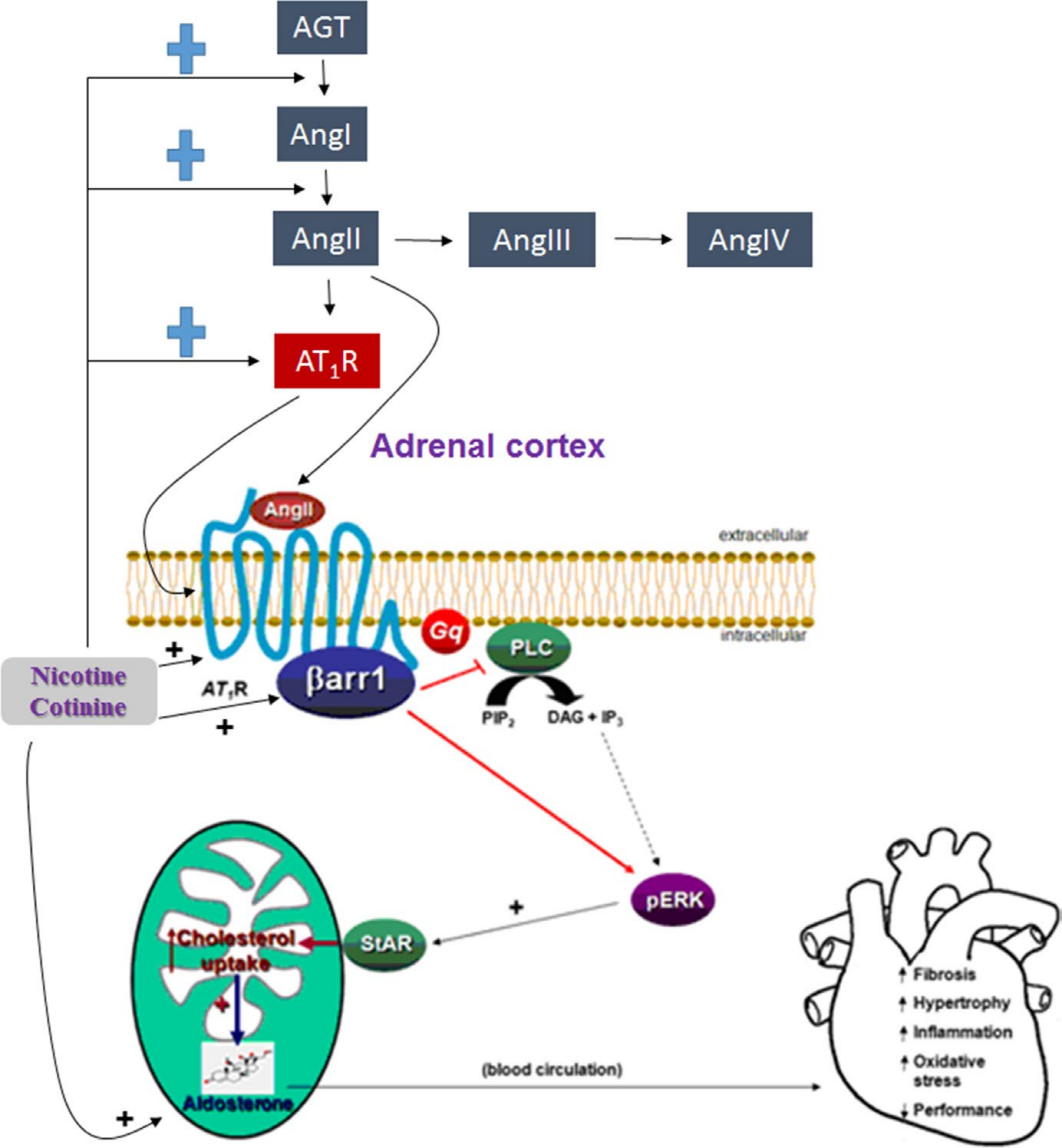
The effect of nicotine/cotinine on RAAS components and on downstream signaling of AngII in adrenocortical cells leading to aldosterone production. The elevated RAAS activity and the putative upregulation of adrenal βarrestin1 (βarr1) by the tobacco compounds nicotine/cotinine are schematically illustrated. AGT: Angiotensinogen; AngI: Angiotensin I; AngIII: Angiotensin III; AngIV: Angiotensin IV; “+” denotes upregulation, that is nicotine/cotinine upregulate AGT to AngI conversion (mediated by renin), AngI to AngII conversion (mediated by angiotensin converting enzyme‐1), and the expression (both mRNA & protein) levels of AT_1_R. Nicotine/cotinine also putatively upregulate adrenocortical βarr1 expression. See text for more details and for all other acronym descriptions

Aldosterone is a mineralocorticoid produced and secreted by the cells of the zona glomerulosa of the adrenal cortex, in response to either elevated serum potassium levels or to AngII, acting through its type 1 receptors (AT_1_Rs), which are endogenously expressed in the adrenocortical zona glomerulosa (AZG) cells.^8^, ^24^ AT_1_Rs belong to the superfamily of G protein‐coupled receptors (GPCRs), and, upon agonist activation, couple to the G_q/11_ family of G proteins,^24^ thus activating the classical phospholipase C (PLC) pathway, which produces the second messengers diacylglycerol (DAG) and 1`,4`,5`‐inositol trisphosphate (IP_3_) (Figure 1).AngII stimulates aldosterone production in AZG cells by binding the AT_1_R, which leads to a G_q/11_‐mediated phosphorylation and activation of the extracellular signal–regulated kinases ERK1/2, members of the mitogen‐activated protein kinase (MAPK) superfamily of kinases^25^ (Figure 1). ERKs, in turn, induce the transcriptional upregulation of the Steroidogenic Acute Regulatory (StAR) protein, a steroid transport protein responsible for the mitochondrial uptake of cholesterol, the precursor of all adrenal steroids^25^ (Figure 1). This procedure represents the first and rate–limiting step of the adrenal steroid biosynthesis, which, specifically in the AZG cells, results in aldosterone synthesis and secretion^8^ (Figure 1).

Over the past few years, however, the AT_1_R, among numerous other GPCRs, has been shown to also signal through G protein–independent pathways.^26^ The protein scaffolding actions of the universal receptor adaptor proteins βarrestin1 and ‐2 (also known as arrestin‐2 and ‐3, respectively), originally discovered as terminators of GPCR signaling following phosphorylation of these receptors by the GPCR‐kinases (GRKs), play a central role in mediating G protein–independent signal transduction by these receptors.^25^, ^27^ More specifically, the AT_1_R, like most GPCRs, upon its agonist activation, is subject to the process of agonist–induced desensitization, which is initiated by its phosphorylation by a serine/threonine kinase family termed GPCR‐kinases (GRKs), followed by βarrestin binding to the receptor that prevents further G protein activation.^25^, ^27^ Subsequently, βarrestin usually targets the receptor protein for internalization; thus, decreasing the number of functional receptors available at the membrane. Once internalized, the receptor is subject to either protein degradation in lysosomes, a process that decreases the total cellular number of receptors and is called receptor downregulation. Alternatively, the receptor gets dephosphorylated and recycles back to the plasma membrane (resensitization).^25^, ^27^ We now know that the two βarrestins are also universal signal transducers for the GPCRs, initiating their own wave of signal transduction from a receptor, once they have physically “uncoupled” it from its cognate G proteins.^26^ Over the past several years, we have shown that βarrestin1, which is by far more abundant than βarrestin2 in the adrenal glands of most species, including humans and rodents, is a crucial mediator of AT_1_R signaling to aldosterone production and secretion, both under physiological conditions and in the context of cardiovascular diseases associated with hyperaldosteronism (high circulating aldosterone levels), such as post‐heart attack heart failure.^28^, ^29^, ^30^, ^31^, ^32^, ^33^ The molecular signaling mechanism underlying this crucial role of βarrestin1 in adrenal aldosterone production is schematically depicted in Figure 1. Specifically, βarrestin1, after uncoupling the agonist–activated adrenal AT_1_R from G proteins, induces activation of ERK1/2 on its own, independently of G proteins. As in the case of G_q_ protein activation, this also leads to StAR upregulation, increased cholesterol uptake by mitochondria, and ultimately, increased synthesis and release of aldosterone by the AZG cell^34^ (Figure 1). Therefore, the salient therapeutic implication of this physiological role of adrenal βarrestin1 is that a drug targeting the AT_1_R (such an ARB‐angiotensin receptor blocker, an AT_1_R antagonist) needs to block both the G protein– and the βarrestin1–dependent pathways equally well in the adrenals, in order to effectively suppress aldosterone production and lower its blood levels, which would also help ameliorate tobacco–related heart disease.

## 3 TOBACCO, ALDOSTERONE AND CARDIAC DYSFUNCTION: FOCUS ON MITOCHONDRIA

The increased activation of the different components of the RAAS are well–known inducers of unbalanced energy production, with the consequent increased production of ROS (reactive oxygen species) and oxidative stress.^35^, ^36^, ^37^ Many components of cigarette smoke can induce oxidative stress in their own right and the potential contribution by nicotine and aberrant RAAS activity is unclear. In any case, the main source of ROS production in cardiomyocytes is mitochondria, specifically the different complexes of the Electron Transport Chain (ETC).^38^, ^39^ One of the first sensors of the cellular damage exerted by increased ROS is mitochondrial DNA (mtDNA), which is extremely sensitive to increased oxidative damage. Different models of cardiovascular disease have shown increased levels of damaged mtDNA.^40^, ^41^

**Figure 2:**
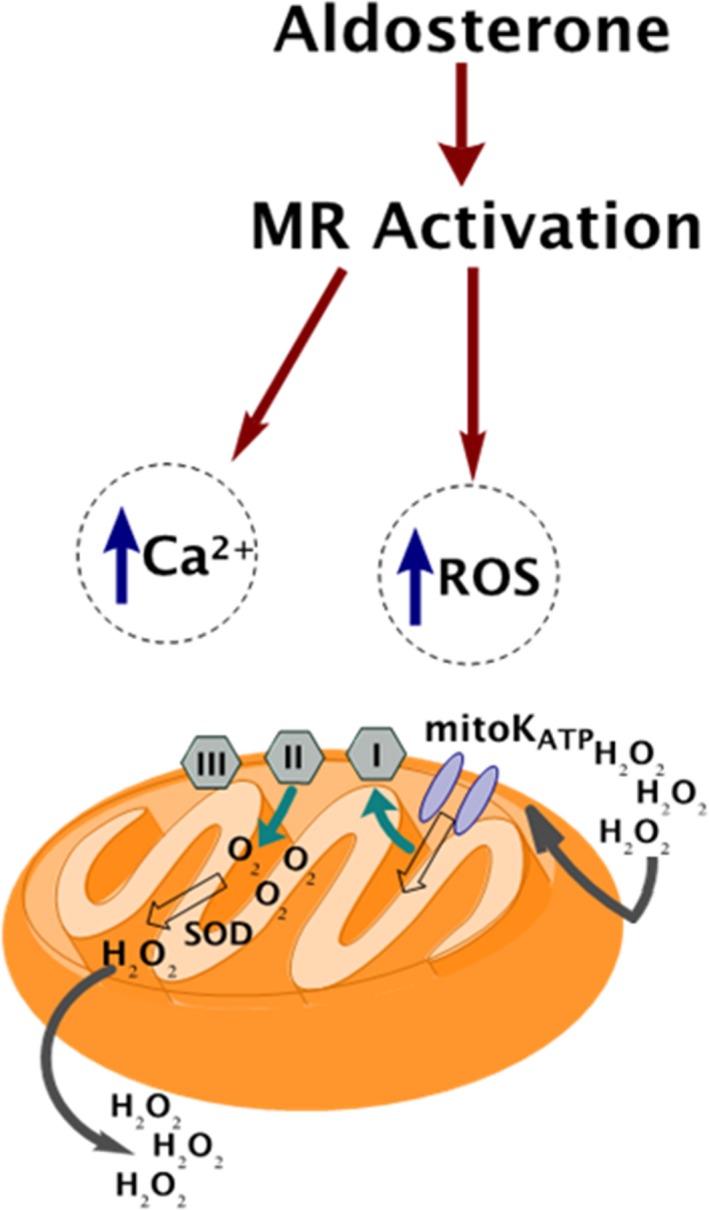
Major mitochondrial effects of aldosterone, through its mineralocorticoid receptor (MR), in the tobacco–exposed heart. Tobacco–elevated aldosterone increases oxidative stress and impairs calcium homeostasis via the MR, causing mitochondrial dysfunction in cardiomyocytes. SOD: Superoxide dismutase. See text for more details and for all other acronym descriptions. Adapted from 48

One of the main effects of the aldosterone–mediated cardiac dysfunction in smokers is the dysfunction of mitochondria within the cardiovascular tissues 42 (Figure 2). In the specific case of aldosterone, its effects on mitochondrial dysfunction are not clear. On one hand, it has been shown that this hormone exerts an A‐kinase anchor protein–mediated positive effect on mitochondrial function in a model of human cardiac fibroblasts.^43^ However, on the other hand, working in different systems in which aldosterone plays a crucial role (ie renal proximal tubular epithelial cells and podocytes) mitochondrial dysfunction has been described.^16^

Increased levels of ROS cause a proinflammatory phenotype, present not only in the vascular sites, but also in the nonvascular ones.^44^ Moreover, long–term exposure to increased ROS has been reported to induce dysfunction in the cardiac tissue, fibrosis and ultimately, mitochondrial–dependent apoptosis.^45^ Interestingly, most of this oxidative damage has been observed not only in active smokers, but also on secondhand ones.^46^, ^47^

The molecular mechanism driving to increased ROS production has not been fully elucidated yet. The induction of the ATP–dependent potassium channel opening, which will induce increased production of ROS, has been proposed as one of the plausible triggers of this process. After this induction, ROS will activate specific membrane transporters, as is the case of the sodium/hydrogen exchanger or the sodium/bicarbonate, by stimulating the ROS–sensitive MAPK cascade.^48^ Interestingly, the bibliography shows that these processes increase cardiac contractility and probably, cardiac hypertrophy too.^48^ Importantly, ROS are not only deleterious molecules, but they also have a crucial role as signaling molecules, mediating different intracellular pathways.^49^ Specifically, the superoxide anion and hydrogen peroxide have been proposed as key secondary messengers of RAAS in different cardiac functions.^36^, ^50^ Thus, the unbalance of the production of these molecules will also affect the activation of RAAS and, ultimately, could negatively affect heart function.

Calcium signaling is another important component of cellular survival, involved in many physiological processes.^51^ Elevated levels of cellular calcium are deleterious and they will induce cell death. Thus, calcium handling dyshomeostasis has also been proposed as a mediator of the damage induced by the increasing activation of the RAAS system.^52^ However, the exact mechanism mediating the relationship between increased activation of RAAS and calcium dyshomeostasis is not yet clear. One potential explanation could be the relationship between renin and calcium. In fact, increased concentrations of calcium in plasma are associated with inhibited renin release, through a mechanism involving cyclic AMP formation and activation of L‐type voltage‐gated calcium channels.^53^, ^54^ Moreover, interestingly, this dyshomeostasis is also mediated by increased ROS.^48^ Thus, in this case also, mitochondria play a crucial role, not only in the production of ROS, but also in calcium homeostasis. In fact, these organelles are one of the main regulators of the ion homeostasis within the cell.^55^ The relationship between increased ROS and calcium levels in this situation can be explained by the nonacute response induced by ROS, which involves calcium–activated targets that participate in cardiac hypertrophy and heart failure.^48^ Interestingly, dysfunctional calcium homeostasis is implicated in many different deleterious cardiovascular outcomes.^56^, ^57^, ^58^

Lastly, Vitamin D and the parathyroid hormone (PTH), two of the main components of the cellular calcium homeostasis, are pivotal in another plausible mechanism explaining the relationship between increased RAAS activation and calcium. In fact, a bidirectional and positive relationship between RAAS and PTH has been reported.^59^, ^60^ Both Vitamin D and PTH are calcium–regulatory hormones, playing a crucial role in maintaining skeletal health, mostly through mobilizing calcium and controlling its absorption in different organs.^52^ They are both required to maintain physiological levels of calcium and phosphate in blood, through a complex integration system, whose dysregulation results in a wide variety of diseases and disorders.^61^ Specifically, high levels of PTH and low levels of Vitamin D have been broadly associated with cardiovascular disease and mortality.^62^, ^63^, ^64^

Both increased levels of ROS and calcium dyshomeostasis are triggers of the irreversible opening of the Mitochondrial Permeability Transition Pore (mPTP), especially when they happen simultaneously.^65^ The extent of the pore opening is determined by the matrix calcium concentration, among other factors.^66^, ^67^ The opening of the mPTP induces increased production of ROS, activation of calcium targets participating in cardiac dysfunction and ultimately, cell death.^48^ One of the molecules with a prominent role in regulation of the mPTP is inorganic polyphosphate (polyP). PolyP has been proposed as an activator and a molecular component of this structure in different systems,^68^, ^69^, ^70^ including cardiomyocytes.^70^, ^71^, ^72^ Moreover, it is a key regulator of mitochondrial calcium buffering within the organelle^73^ and it is also involved in controlling ROS production.^74^ Thus, this small molecule could potentially play a big role on the observed RAAS–induced mitochondrial dysfunction. In fact, its pharmacological modulation could represent and extremely innovative and exciting challenge in the study of treatments for cardiac dysfunction.

## 4 **PHARMACOLOGICAL HYPOTHESIS: TOBACCO STIMULATES ADRENAL** β**ARRESTIN1 TO INDUCE HYPERALDOSTERONISM**

Given that AngII induces aldosterone production in AZG cells by binding to its adrenal AT_1_R, which then activates βarrestin1^28^ (Figure 1) and nicotine/cotinine are known to activate RAAS,^13^ promoting AngII actions at its various tissue targets, including the adrenal cortex, tobacco compounds may chronically increase AngII–dependent aldosterone production in AZG cells via adrenal βarrestin1. Therefore, we have hypothesized that chronic nicotine/cotinine (ie tobacco) exposure upregulates adrenal AT_1_Rs and βarrestin1, promoting excessive aldosterone synthesis and secretion from human AZG cells. Thus, adrenal βarrestin1 is a crucial component of tobacco–induced RAAS activation, which contributes to heart disease development/progression.

To determine whether βarrestin1 is involved in tobacco–dependent adrenal aldosterone production, the human AZG cell line H295R, which endogenously expresses the AT_1_R (but not the AT_2_R) and βarrestin1 can be used.^28^ This cell line produces and secretes aldosterone in response to AngII stimulation.^28^ These H295R cells can be treated with various concentrations of tobacco compounds (eg nicotine) for several consecutive days to simulate chronic tobacco exposure. In the end, the cells are challenged with AngII to measure the evoked aldosterone secretion via ELISA. StAR protein expression can be also examined as a marker of aldosterone biosynthesis.

Based on the associated literature, AngII–dependent aldosterone secretion and StAR protein levels should be significantly higher in AZG cells treated with nicotine compared to control, vehicle–treated cells (Figure 1), and nicotine might upregulate βarrestin1 (along with the AT_1_R itself) in AZG cells (Figure 1).

## 5 THERAPEUTIC PERSPECTIVES

Nicotine and cotinine are expected to increase adrenal AngII–dependent, βarrestin1–mediated aldosterone synthesis and secretion both in vitro and in vivo. Elevated aldosterone leads to development of cardiac dysfunction. Therefore, adrenal βarrestin1 inhibition might prove to be a novel therapeutic strategy for prevention or amelioration of tobacco–related heart disease. Aldosterone and its receptor, the mineralocorticoid receptor (MR), have been established for a long time as important molecular culprits of heart failure (and of heart disease, in general) progression. In fact, a very recent study conducted in transgenic mice, demonstrated that, among the adrenal steroids acting on the heart, it is the MR (and not the closely biochemically related glucocorticoid receptor) that promotes cardiac dysfunction and cardiomyopathy, even in the absence of a cardiac insult (eg myocardial infarction or pressure overload).^75^ This work strongly suggests that all of the effects of aldosterone in the heart (mediated by the MR) are harmful, whereas glucocorticoids, including the endogenous glucocorticoid hormone cortisol, may actually exert beneficial or protective effects in the myocardium. Therefore, aldosterone is definitely one of the hormones whose levels need to be suppressed for therapeutic purposes in heart disease. Given various reports that this hormone oftentimes acts in an MR–independent manner, which circumvents the actions of MR antagonist drugs, an approach to suppress its cardiotoxic actions is the direct inhibition of its production in the adrenal cortex.

Over the past decade or so, we and others have established adrenal βarrestin1 blockade as a very effective way of achieving precisely that, ie suppression of adrenal aldosterone production.^10^ Adrenal βarrestin1 blockade is obviously feasible via gene therapy to knock down the protein or even delete its gene specifically from the adrenal glands (eg via CRISPR/Cas9–mediated gene deletion). However, the most realistic approach to block adrenal βarrestin1 clinically would be pharmacologic blockade of the adrenal AT_1_R, with a very potent antagonist (angiotensin receptor blocker‐ARB drug) that effectively suppresses both G_q/11_ protein– and βarrestin1–dependent aldosterone production pathways in AZG cells, such as candesartan or valsartan.^30^, ^32^ Alternatively, the βarrestin1–mediated signaling to aldosterone synthesis could be targeted with barbadin, a compound that was recently identified as an inhibitor of βarrestin–dependent internalization and signaling.^76^ However, regardless of the approach chosen, adrenal–specific targeting is of paramount importance, since, unlike the adrenal cortex, where they converge on stimulation of AngII–dependent aldosterone production, G proteins and βarrestins can have different and opposing signaling effects in other organs/tissues even within the cardiovascular system (eg heart, vasculature, platelets, etc).^27^

## 6 CONCLUSIONS

Smoking kills 6 million people each year, with 480 000 deaths in the US alone. Although current trends suggest that the prevalence of smoking is declining, the overall decline is slow, and the use actually is increasing in many low– and middle–income countries. Global health costs are expected to rise over the next decade, especially with the growing popularity of novel nicotine–delivery devices, such as e‐cigarettes, flavored and unflavored vapor nicotine–containing liquids, etc, which are highly engineered, pleasurable, and rapid in their delivery of the highly addictive nicotine. Despite significant advances in the understanding of the pathophysiology of tobacco–related heart diseases, tobacco–related products have continued to evolve faster than scientific knowledge of their biological effects. It is thus imperative to devise novel, preventative and therapeutic strategies to protect smokers against tobacco`s devastating effects on their cardiovascular health.

Since aldosterone plays an important detrimental role in promoting tobacco`s cardiotoxicity, which includes maladaptive changes in cardiac inflammation, structure, energetics, mitochondria, and in a plethora of other cardiac parameters, we have developed a hypothesis that nicotine and its metabolites actually promote adrenal aldosterone production via upregulation of the AngII receptor‐adapter protein/signal transducer βarrestin1. Thanks to the tremendous progress in the field of βarrestin–dependent signal transduction research over the past 20 years or so, this protein can now be targeted (blocked) pharmacologically in a tissue–specific manner. Therefore, if our herein outlined mechanistic hypothesis proves true in vitro and, more importantly, in vivo, adrenal βarrestin1 blockade may one day find its place in the clinically available armamentarium of tobacco–related heart disease prevention and treatment.

## DISCLOSURES

None declared.
